# The use of artificial songs to assess song recognition in imprinted female songbirds: a concept proposal

**DOI:** 10.3389/fpsyg.2024.1384794

**Published:** 2024-09-04

**Authors:** Hiroharu Makioka, Rebecca N. Lewis, Masayo Soma

**Affiliations:** ^1^Biosystems Science Course, The Graduate School of Life Science, Hokkaido University, Sapporo, Japan; ^2^JSPS International Research Fellow, Department of Biology, Faculty of Science, Hokkaido University, Sapporo, Japan; ^3^Department of Biology, Faculty of Science, Hokkaido University, Sapporo, Japan

**Keywords:** sexual selection, learning, synthesizing song, preference, Java sparrow, *Padda oryzivora*

## Abstract

We propose an experimental paradigm to examine acoustic features responsible for song preference and recognition in songbirds. Song preference in female songbirds is often influenced by early song experience. That is why several Estrildid species, including our subject species, the Java sparrow (*Padda oryzivora*), are known to show an imprinted preference for their father’s songs. After confirming that Java sparrow females preferred their father’s song compared to non-imprinted through song playbacks (first step), we repeated the playback tests in the same subjects using synthesized stimuli (second step). To create synthesized stimuli, we removed all the complex frequency modulations and subharmonics from song notes that we used for the first step playback tests to see the effect of spectrometric features on song recognition. The results indicated that females showed higher rate of calling towards synthesized father song stimuli, suggesting that the macroscopic patterns would play more important roles in song recognition than the microscopic acoustic features. Although we looked at spectrometric features and father-imprinted song preference in this study, similar testing can be applied in many ways to test preference for local dialects or subspecies-specific songs.

## 1 Introduction

In order to elucidate the evolution of birdsong, it is crucial to understand the cognitive and social processes of song communication. The diversity of acoustic structures of birdsong is an evolutionary outcome of reproductive competition and mate attraction (Searcy and Andersson, 1986; Catchpole and Slater, 2003), where unlike morphological sexual traits, learning can mediate both cultural inheritance of songs and the development of song preferences (Slater, 1986; Beecher and Brenowitz, 2005; Williams, 2021). In general, when males sing to attract females, male song learning capacity can be assessed by females – this is the commonly accepted idea of how intersexual selection would play a role in the evolution of vocal learning ability in males. However, testing female song recognition and preference is challenging for two reasons, (1) difficulty in controlling for early social experience that can affect female perception (Miller, 1979a; Riebel, 2000; Holveck and Riebel, 2010), and (2) limitations inherent in each of existing test protocols, which both we will elaborate below (see also Table 1). Given these challenges, we would like to propose a new experimental paradigm to examine acoustic features responsible for song preference and recognition in songbirds in this paper.

**Table 1:** Comparison of behavioral test methods commonly used for the investigation of song discrimination or preferences.

	Belief method	Female response	Bias caused by preferences for species or imprinted songs	Possible use of artificial song. Modification of -	
				macroscopic aspects	microscopic aspects
Discrimination test (operant conditioning)	Using either food rewards or darkness punishment, subjects are trained to respond to song A (Go) not B (non-Go).	Operant-conditioned behaviors^1^^,^^2^^,^^3^	Possible^12^	OK^1^^,^^2^	OK^3^
Preference test (operant conditioning)	Using song stimuli as rewards, subjects are trained and tested to see whether they preferentially listen to song A or B.	String pulling^4^ Key pecking^5^^,^^6^ Perch visiting^7^	NA (bias can be a part of preference)	OK^7^	Possible but caution needed as modified songs can sound unnatural (see Figure 1)
Songs Playback test	Subjects are exposed to the playbacks of song stimuli.	CSD (Copulation Solicitation Display)^8^ Call back^8^^,^^11^ Phonotaxis^9^^,^^10^	NA (bias can be a part of preference)	OK	Possible but caution needed as modified songs can sound unnatural (see Figure 1)
This study		Call back^11^	Propose to control for it	OK	OK

1 Okanoya et al. (2000).

2 Lawson et al. (2018).

3 Watanabe et al. (2006).

4 Barr et al. (2021).

5 Riebel (2000).

6 Salvin et al. (2018).

7 Wei et al. (2022).

8 Fujii and Okanoya (2022).

9 Fujii et al. (2021).

10 Loning et al. (2023).

11 Lewis et al. (2024).

12 Caspani et al. (2020).

In general, female song preference is said to emerge early in life due to sexual imprinting on adult conspecific males (Marler, 1990; Fujii et al., 2022). This can lead either to broad scale preferences, such as local dialect preference (O’loghlen et al., 1995; Searcy et al., 2002), or smaller scale preferences, including a controversial phenomenon where females prefer their father’s songs. Preference for father’s song has been frequently reported in Estrildid finches, including zebra finches (*Taeniopygia guttata*: Miller, 1979a,b; Riebel, 2000; Riebel and Smallegange, 2003), Bengalese finches (*Lonchura striata* var. *domestica*: Kato et al., 2010; Fujii et al., 2021), and Java sparrows (Lewis et al., 2024). This is not only puzzling, considering the adverse effect of inbreeding depression (Bolund et al., 2010), but also making it hard to test female song preference (Table 1). There is not yet a concrete answer to the question of whether females prefer males that only accurately learned songs or those that can learn to sing more variable/complex songs including zebra finches. It is possible that females prefer both, but it should be noted that making a perfect copy of poor song model would sacrifice song complexity (cf. Soma et al., 2009). What is clear from previous studies is that males tend to imitate their tutors’ songs very well, especially in non-territorial species in captivity (zebra finch: Tchernichovski and Nottebohm, 1998; Holveck et al., 2008; Java sparrow: Lewis et al., 2021; Lewis et al., 2023), leading to a high degree of song-notes sharing and note similarity between tutors and tutees (Brainard and Doupe, 2002; Lewis et al., 2021).

**Figure 1:**
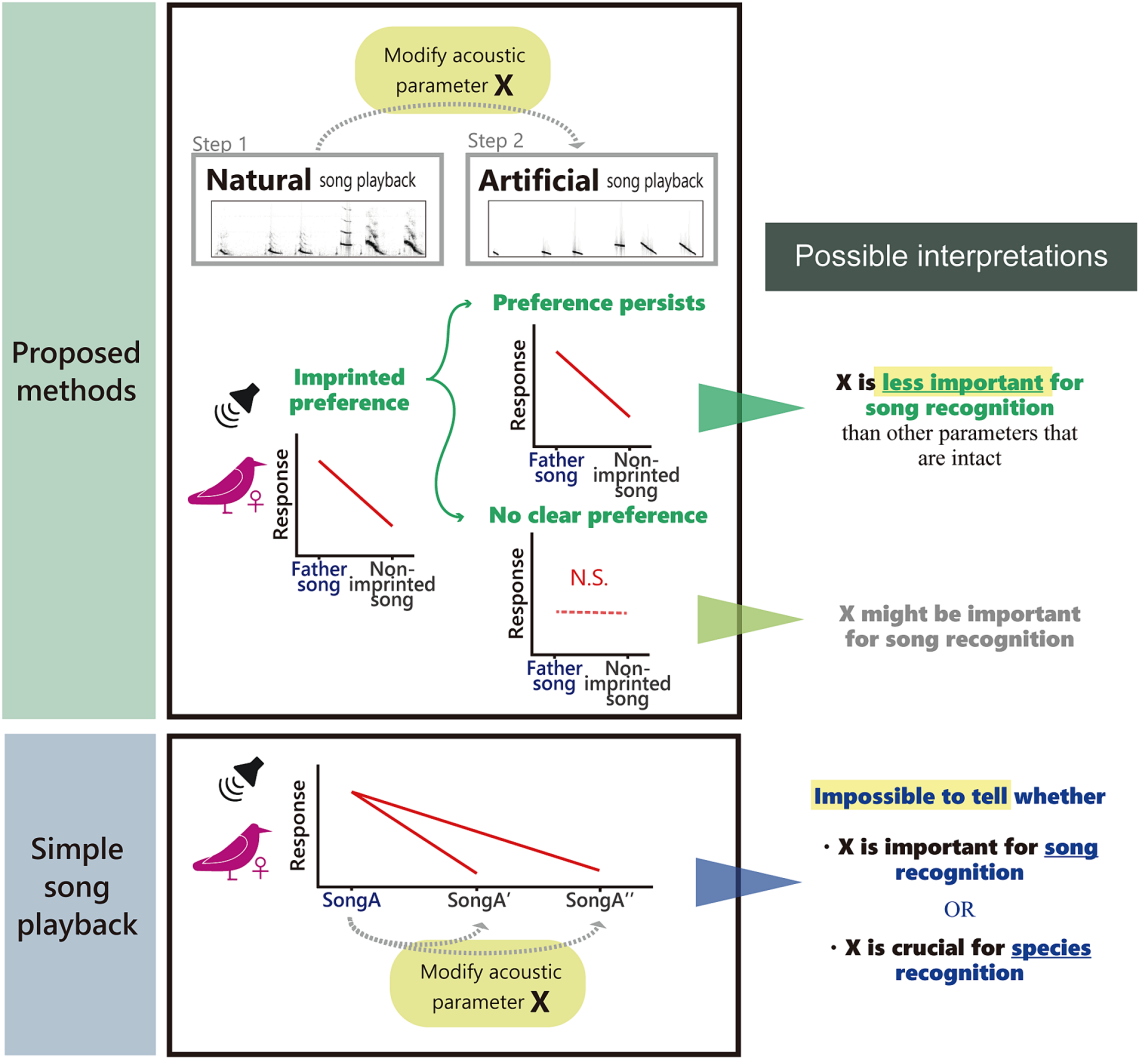
The overview of our experimental paradigm. Unlike simple song playback experiments, two-step playback experiments using females sexually imprinted with father songs and modified father and control songs can shed light on relative importance of acoustic parameters important for song recognition and preference.

It is technically challenging to test female preference for song learning accuracy. One previous study reported that zebra finch females cannot generalize their preference for their father’s songs to their male siblings’ songs (Riebel and Smallegange, 2003), implying that siblings’ songs were not accurate enough copies of the father’s song. So far, it is still in debate how much and on what basis females generalize their imprinted song preference (Wei et al., 2022). In order to understand female song preference and discrimination, macroscopic aspects of songs, such as syllable orders or repertoires, can be experimentally modified for playback tests to determine females’ responses (Vernaleo and Dooling, 2011; Lawson et al., 2018; Wei et al., 2022). However, changes in microscopic acoustic structures, such as frequency modulation, harmonics, or amplitude modulation, are harder to study. This is because artificially modified song notes may sound unnatural to subject birds (Table 1). Therefore, if females respond less to artificial stimuli, it could be because the modification affected the attractiveness of songs, but it is also possible that females do not recognize these songs as conspecific (Figure 1). For example, canaries (*Serinus canaria*) showed fewer responses to the artificial sexy phrases with modified amplitude modulation than natural ones (Pasteau et al., 2012, but see Drăgănoiu et al., 2002). What is already known for song recognition in females is that they prefer songs of mates, kin, neighbors or conspecifics (Marler, 1990; Beecher and Brenowitz, 2005; Fujii et al., 2022). Generally, female songbirds seem to discriminate between the songs of their mate and other males (zebra finch: Miller, 1979b; song sparrow, *Melospiza melodia*: O’loghlen and Beecher, 1997), local dialect (song sparrow: Harris and Lemon, 1974; white-crowned sparrow, *Zonotrichia leucophrys*: O’loghlen et al., 1995; zebra finch: Wang et al., 2022; Loning et al., 2023), and species-specific songs (zebra finch: Clayton, 1990). However, it is unclear which acoustic characteristics are important for their recognition.

**Figure 2:**
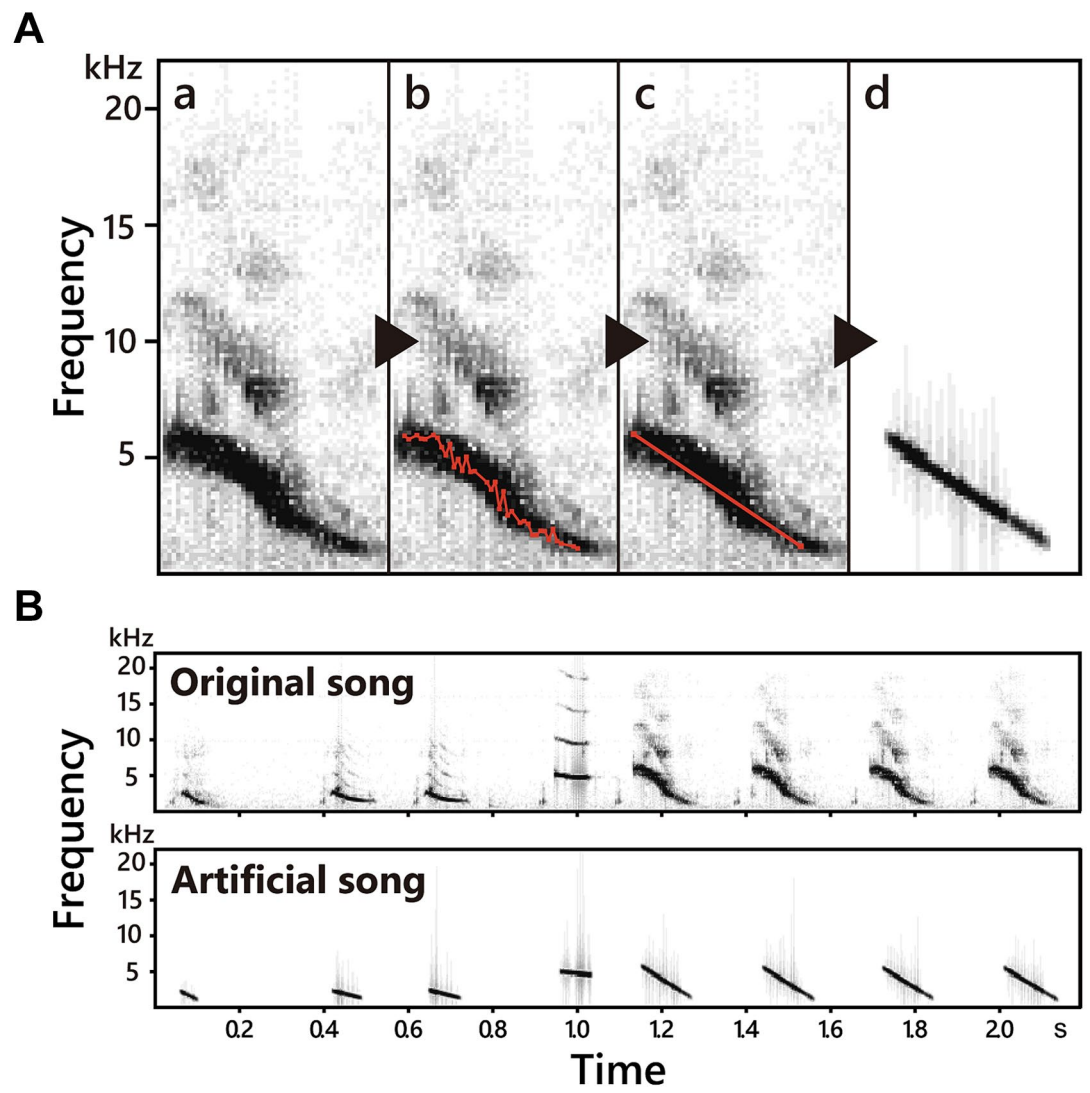
The methods of synthesizing song notes **(A)** and an example of original and synthesized song **(B)**. In this study, we modified spectral characteristics of song notes to obtain notes with simpler frequency structures. Firstly, we scanned frequency and drew contour of notes **(a–b)**, and then removed detailed frequency up-downs **(c)** to obtain notes with linear frequency modulation and without harmonics **(d)**.

Given these challenges, we propose an experimental paradigm to examine acoustic features responsible for song preference and recognition in songbirds. As mentioned earlier, females of several Estrildid species show an imprinted preference for father or familiar song that they heard early in life (zebra finch: Marler, 1990; Bengalese finch: Fujii and Okanoya, 2022), which was also confirmed in our subject species, the Java sparrow (Lewis et al., 2024). If we confirm that females respond more towards imprinted over non-imprinted songs, then we can ask them what acoustic features they base their response on by using artificial song stimuli. To this end, we designed a two-step playback experiment (Figure 1). In the first step (Lewis et al., 2024), playback tests were conducted using a pair of natural songs (father vs. non-imprinted) to confirm imprinted preference. In the second step, which is presented in this manuscript, we synthesized artificial Java sparrow songs based on natural ones, and repeated the playback test again using the same female subjects and the same combination of songs. Although we used pair-wise playback to assess preference, it should be noted that this protocol can be used with any preference measure, e.g., string-pulling test or phonotaxis for two sets of song stimuli (e.g., Holveck and Riebel, 2007; Barr et al., 2021). We also stress that the synthesizing method we used is just an example. In this study, we focused on the spectral characteristics of song notes when creating artificial songs. Specifically, we removed all complex frequency modulations and subharmonics from natural notes to obtain tonal notes with linear frequency modulation, whilst keeping temporal patterns intact (Figure 2). If the previously observed preferences within the natural song stimuli pair is evident in artificial stimuli pair as well, we can conclude that the modifications made to create the artificial stimuli are less important for song recognition or preference than the other acoustic structures that remained intact. If the preference disappears for artificial stimuli, it is possible that our modification changed crucial aspects of the song (Figure 1).

## 2 Materials and methods

### 2.1 Playback experiments

Here, we propose a two-step playback experiment, firstly using natural songs (Lewis et al., 2024) to confirm females’ preferences and then using artificial songs to test specific song features (current manuscript; Figure 1). For the second step experiments, presented here, we used the same Java sparrows (*Padda oryzivora*) females as Lewis et al. (2024), which were confirmed to show preference for father songs. Playback experiments for the first and second steps occurred 2 years apart. Due to the life span of the species, only four Java sparrow females (5–6 years old) were available to complete the second step playback experiments. Females were housed in single-sex cages (91 × 44 × 44 cm or 54 × 44 × 44 cm) with 4–8 females except during the experimental period or breeding. None of the subjects had visual interaction with other birds in adjacent cages, but they were able to hear other birds’ songs and see individuals in cages opposite. Although all the subjects had prior experience of breeding (i.e., nesting, laying eggs), their mating partner’s songs were not used as stimuli. The subjects were given mixed seed (millet, foxtail millet, Japanese millet, and canary seed), crumbled oyster shell, water, and vegetables daily, and egg coated mixed seed during the experimental period, and were in 12-h light and 12-h dark cycles (lights on between 8:00 and 20:00 JST). The temperature was maintained at around 25–30°C and humidity was maintained at around 40–60%.

We conducted the playback experiments for artificial songs based on the protocol for natural songs (for details, see Lewis et al., 2024). Briefly, subjects were isolated into the experimental cages (37 × 25 × 19 cm) in a sound attenuated chamber 3 days before the tests for habituation. The song stimuli were played back to the subjects by a speaker (Micro wireless, JBL, California, USA), and the subject’s behaviors were recorded with a video camera (Q4n, ZOOM, Tokyo, Japan). Each subject experienced one playback session per day with two-day intervals, and in total four sessions, two with father vs. non-imprinted, and the other two with the same song lineage (non-father) vs. different song lineage (Supplementary Figure 1). As songs are culturally inherited from fathers to sons in the lab population (Lewis et al., 2021), family members share a similar song type, called a song lineage, which is audially and visually identifiable. Song lineage is independent from genetic relationships as most individuals were reared by foster parents (Lewis et al., 2021). In this study, the term ‘non-imprinted songs’ does not mean that females have never heard them, but refers to song types sung by individuals belonging to a different song lineage to the female’s social father (Lewis et al., 2021; Lewis et al., 2023). As found in Lewis et al. (2024), the four subjects lacked preference for non-father songs from the same song lineage in the natural song experiments (Lewis et al., 2024), so we focused on father vs. non-imprinted experiments only here (see Supplementary Figure 2). Each father vs. non-imprinted session consisted of four trials, alternating between the father’s song and non-imprinted songs (Supplementary Figure 1). Within each trial, songs were played back at a constant rate (1 stimulus every 10 s) for a total of 2 min, meaning that each trial included 12 repetitions of songs of each male (4 repetitions of 3 different songs in alternation), and trials were separated by 30-s intervals (Supplementary Figure 1). The playback order of the stimuli in the trials and sessions was counterbalanced between subjects in the first step, and the orders of each subject’s experiment in the second step followed the orders of the first step, respectively.

### 2.2 Song stimuli

The playback song stimuli (both father and non-imprinted songs) were modified from the songs of previous experiments (Lewis et al., 2024) using Avisoft-SASLab Pro (ver. 5.3.02, 2022, Avisoft Bioacoustics). Songs were recorded and archived in our lab (44.1 kHz sampling rate, 16-bit resolution, and on average, 29.48 song notes and 5.16 s. duration per song, Supplementary Table 4). In order to simplify the song notes, the frequency modulation of the notes was adjusted to be linear, and the sub-harmonics of the notes were unified into the fundamental tone without altering the amplitude modulations, rhythms, and note sequences (see Figure 2A for detailed methods). Specifically, we used the function “scan frequency contour” of Avisoft-SASLab Pro, with which each song was sectioned and illustrated as single lines and dots on the spectrogram (Figures 2Aa,b), and then dots except the beginning and end of the sections were removed (Figures 2Ac,d).

### 2.3 Behavioral and statistical analyses

As behavioral indices of female preference, we counted the number of calls produced by each female subject during each playback trial based on recorded video data. The behavioral coding was conducted by an observer (HM) manually. Calling response is one of the most commonly taken behavioral parameters in song preference test, and is known to correlate with other preference-associated behaviors, such as copulation solicitation display or phonotaxis in related species (Clayton, 1988; Dunning et al., 2014; Fujii et al., 2021). Also in Java sparrows, calls function in sexual and social communication (Goodwin, 1982). Additionally, we also measured the frequency of hops, bill wiping, and fluffing shown during each trial. Both hopping and bill wiping are behavioral elements of Java sparrow’s mutual courtship interaction (Soma and Iwama, 2017). Fluffing posture was taken as an indicator of song preference in a previous study in a related species, the zebra finch (Vyas et al., 2009).

All analyses were performed with R ver. 4.4.0 (R Core Team, 2024). To examine whether the females showed more responses (i.e., calling, hopping, bill wiping and fluffing) towards synthesized father songs than to synthesized non-imprinted songs, we conducted a series of generalized linear mixed models (GLMMs), one for each behavior. We included stimulus type (father vs. non-imprinted), the order of trial within the session (1–4), and the order of the session within the experimental period (1–4) as fixed effects, and the subject female’s ID, the song owners’ ID, and their interaction as random effects of the models to avoid pseudo-replication and consider individual specificity. Females did not always respond to stimuli, which may result in models being zero-inflated. We checked for zero inflation using the check_zeroinflation function from the performance package (Lüdecke et al., 2021) and adjusted analyses accordingly. Therefore, the number of calls, hops and bill wiping were analyzed with zero-inflated GLMMs using the glmmTMB package including the zero-inflation formula (Magnusson et al., 2017). Zero-inflated models consist of two parts, binomial (0–1) and count processes, which are called zero-inflation and conditional models, respectively. As statistical outcomes of these models, we focused on the conditional models. The number of fluffing was analyzed with a GLMM using the glmmTMB package, without the zero-inflation formula. All variables were included in the zero-inflation formula of the model. If the model did not run correctly with all variables included, the variables were selectively removed, comparing the AICs (Akaike, 1998) of the models. In addition, to confirm not to be affected by a specific individual’s response, calling response was also analyzed at the individual level in a similar fashion. To examine whether the differences between responses to father and non-imprinted songs are consistent in the first and second steps of the experiment, we analyzed the combined datasets of calling responses of the four subjects from both steps of the experiment with zero-inflated GLMM. We used the same model described above, with the addition of an interaction term of the first vs. second steps (artificial vs. natural songs) and father vs. non-imprinted songs.

## 3 Results

**Figure 3:**
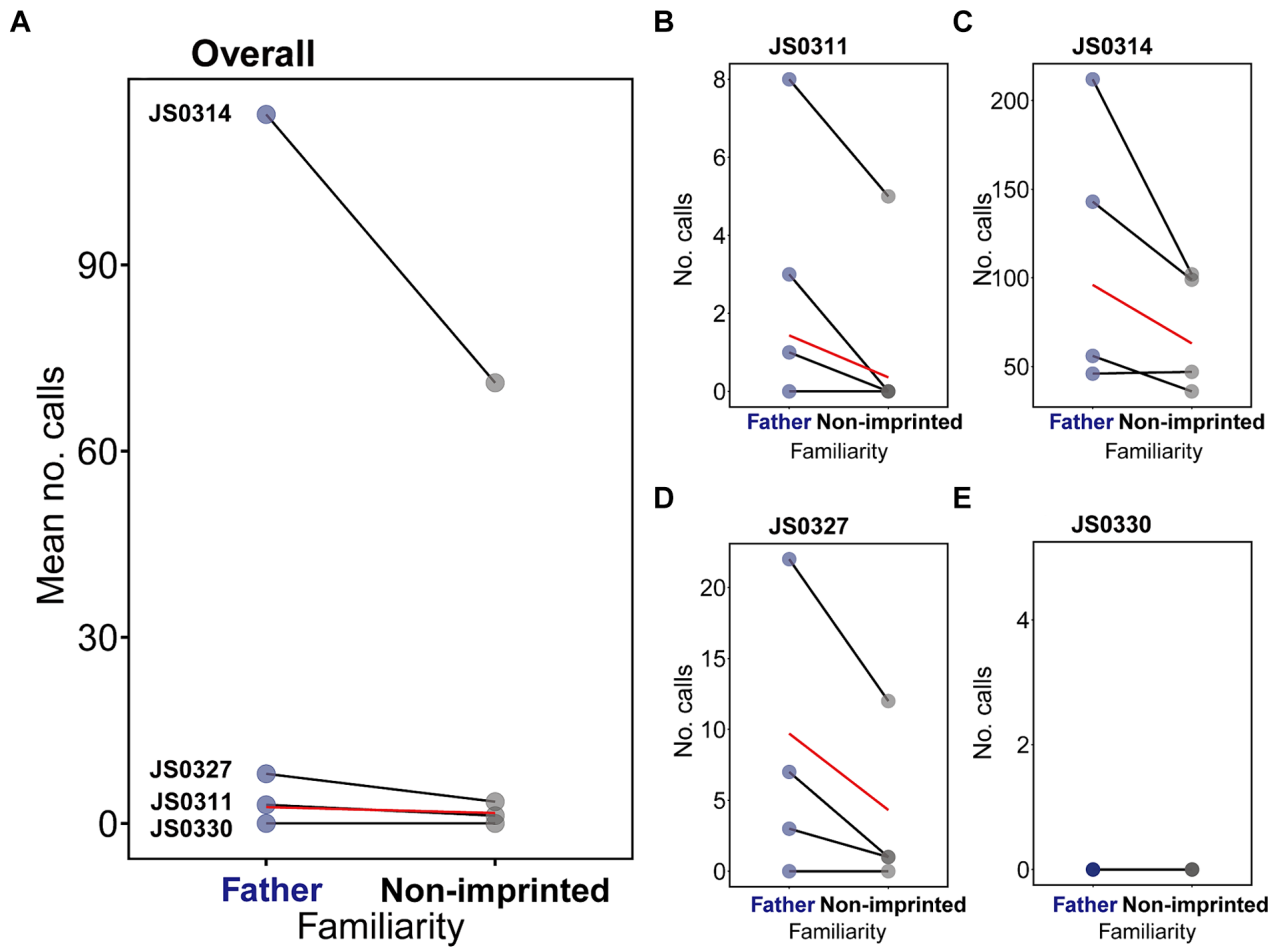
The results of calling responses to the artificial father and non-imprinted songs. **(A)** shows the average number of calls per trial shown by four subject females, where each connected dots correspond to each subject, and the red line indicates the effect of the familiarity on the statistically significant difference. **(B–E)** show the calling responses shown by each subject in each trial, where the line connects the same order trials.

The subjects showed more calling responses to the artificial father than to the artificial non-imprinted songs (Overall subjects: zero-inflated GLMM, β = −0.43, *p* = 0.001, Figure 3A, Supplementary Table 1), which was also evident in each subject data (JS0311: GLMM, β = −1.97, *p* = 0.007, Figure 3B; JS0314: GLMM, β = −0.42, *p* < 0.001, Figure 3C; JS0327: zero-inflated GLMM, β = −0.81, *p* = 0.016, Figure 3D; Supplementary Table 2) except for subject JS0330 that did not show any responses to songs (Supplementary Table 2). Calling responses increased with session order (zero-inflated GLMM, β = 1.02, *p* < 0.001; Supplementary Table 1; Supplementary Figure 3), which varied among individuals (Supplementary Table 2), and decreased with trial order (zero-inflated GLMM, β = −0.12, *p* = 0.036, Figure 3E; Supplementary Table 1; Supplementary Figure 4). Considering that JS0330 showed no response throughout all the trials, we also re-ran the model excluding data from JS0330. Even after excluding JS0330, who did not call throughout the study, the familiarity effect on calling was significant (GLMM, β = −0.42, *p* = 0.014). However, for other behavioral parameters, the difference was not statistically significant (hop: zero-inflated GLMM, *p* = 0.942; fluffing: GLMM, *p* = 0.412; bill wiping: zero-inflated GLMM, *p* = 0.347; Supplementary Table 1; Supplementary Figure 5).

When we analyzed the combined dataset of calling responses from the first and second steps, there was no statistically significant effect of the interaction of familiarity × step (zero-inflated GLMM, *p* = 0.310; Supplementary Table 3), and calling response was greater for artificial songs (zero-inflated GLMM, β = −2.46, *p* < 0.001).

## 4 Discussion

In this paper, we aim to propose a new concept of methodology, which is two-step playback tests to assess song recognition underlying preference in female songbirds focusing on detailed spectrometric features. The results reported here are preliminary with very small sample size and cannot validate the exact playback protocol. Even so, we believe that the combination of two-step preference test and acoustic modification of stimuli would open a new window for understanding the role of female song recognition in song evolution. Regardless of the playback protocols of preference tests or acoustic modifications, the general study design proposed here can be applied to many songbird species (Table 1). However, our proposed paradigm is not without problems. As it is a two-step experiment, it takes time, making it difficult to keep the subjects’ condition and motivation constant throughout the whole experiment. This may have contributed to a lack of calling response in one of the subjects. More fundamental questions regarding this paradigm are the time period over which females continue to show song preference shaped in early life, and the strength of these preferences. Using our proposed paradigm or any other, it would be worthwhile to look into the repeatability of song preference both within and between females. Even so, our proposed methods would be useful for separating song features that otherwise covary.

Our proposed methods can potentially assess female preference for detailed spectrometric features, which tended to be neglected in favor of song/syllable repertoire and sequence in past song preference research (e.g., Bengalese finch: Kato et al., 2010; zebra finch: Woodgate et al., 2011; Wei et al., 2022). By using synthesized song stimuli and female Java sparrows for which imprinted preference for father songs was confirmed, we were able to reveal the relative importance of spectrometric features (i.e., harmonics, detailed frequency modifications) that played a role in their song recognition (Table 1). Subject females showed similar tendencies of calling responses toward both natural (Lewis et al., 2024) and artificial song sets, after controlling for the effects of repeated trials and sessions, meaning that the modifications that we made to songs did not have a major impact on the song recognition. In other words, we assume that the synthesized songs retained acoustic features necessary for recognition and preference. It was unexpected that the subjects showed more calling responses in the second step than in the first step (Supplementary Table 3). Probably, it could have been caused by the differences in the physical conditions between the two steps, but we could not rule out the possibility that the subjects might actually preferred the artificial songs. Although we looked at spectrometric features and father-imprinted song preference in this study, similar testing can be done in many ways to explore song preference (Table 1). For example, this testing paradigm can be easily applied to preference for local dialect (Milligan and Verner, 1971; Wang et al., 2022), or (sub) species-specific songs (Clayton, 1990; Whaling et al., 1997; Soha and Marler, 2001).

The present results suggest that the spectrometric acoustic features we modified are not necessary for the Java sparrow females’ song recognition. Generally, it is thought that songbirds recognize songs by detailed acoustic features of notes (syllables), rather than macroscopic song structure (Review in Fishbein et al., 2020). Zebra finches can discriminate motifs consisting of synthesized syllables from those consisting of natural syllables, but cannot discriminate motifs with altered syllable order from those with natural order (Vernaleo and Dooling, 2011; Lawson et al., 2018). In Bengalese finch, males discriminate their own songs by the note order despite individual song notes being locally reversed, on the other hand, detailed acoustic features appear important for discriminating the songs of other individuals (Okanoya et al., 2000; Kato et al., 2010). Considering accurate song learning in male Java sparrows (Lewis et al., 2021), it was unexpected that females recognize songs mainly by macroscopic features. One possibility is that females might pay attention to macroscopic aspects of songs rather than to detailed spectrometric features that can be easily affected by sound attenuation in the distance. Generally, high-frequency components can be attenuated easier than low-frequency components (Evans et al., 1972; Marten and Marler, 1977). Therefore, higher frequency compartments (sub-harmonics) or complex frequency modulations in their songs might be attenuated over long distances. In white-browed warblers (*Basileuterus leucoblepharus*), some song components, such as slow frequency modulation, are degradation resistant and used to transfer information over long distances, whereas more complex components are more susceptible to degradation and used for short-distance information transfer (Mathevon et al., 2008).

When examining female behavioral responses to male song playback, the interpretation of the behavioral changes needs caution, as greater numbers of responses do not always assure mating preference. For instance, a greater number of calls in response to particular song stimuli can be taken as either song preference or social motivation to interact with the singer (e.g., father; Fujii et al., 2022). However, as in the present experiment, observing the difference in the response would be at least useful for testing song recognition. For a similar reason, non-significant differences in some behavioral parameters other than calling in this study did not necessarily mean that females cannot distinguish the two stimulus types. Based on the differences in calling frequency, it is likely that females could discriminate between the two but just did not show differential responses for some behaviors.

## Data Availability

The original contributions presented in the study are included in the article/Supplementary material, further inquiries can be directed to the corresponding author.
