# Supplementary material for: The use of artificial songs to assess song recognition in imprinted female songbirds: a concept proposal
Source: Front Psychol. 2024 Sep 4;15:1384794. doi: 10.3389/fpsyg.2024.1384794 (PMC11408183; doi:10.3389/fpsyg.2024.1384794)
Supplement: Supplementary file 1 [file Table_1.DOCX]

Supplementary Material

**Supplementary Table 1.** The results of GLMMs for each behavior. Zero-inflated models consist of two parts, binomial (0-1) and count processes, which are called zero-inflation and conditional models respectively. The models included all the explanatory variables except when they failed to converge. Some zero-inflation models had to be run without familiarity effect as they could not converge with it.

| Session type | Father vs. Non-imprinted | |  | Same vs. Different song lineage | |
| --- | --- | --- | --- | --- | --- |
| Fixed Effects | Estimate | *p*-value |  | Estimate | *p-*value |
| Call | | | | | |
| Conditional model | | | | | |
| Familiarity (Non-imprinted) | **-0.43** | **0.001** |  | **0.55** | **0.026** |
| Session Order | **1.02** | **<0.001** |  | 0.34 | 0.154 |
| Trial Order | **-0.12** | **0.036** |  | **-0.31** | **0.005** |
| Zero-inflation model | | | | | |
| Familiarity | 0.46 | 0.786 |  | **-** | **-** |
| Session Order | 2.00 | 0.533 |  | 0.39 | 0.691 |
| Trial Order | 3.07 | 0.176 |  | -0.52 | 0.639 |
| Hop | | | | | |
| Conditional model | | | | | |
| Familiarity | 0.02 | 0.932 |  | 0.10 | 0.706 |
| Session Order | **0.71** | **0.039** |  | 0.13 | 0.554 |
| Trial Order | 0.02 | 0.902 |  | -0.01 | 0.965 |
| Zero-inflation model | | | | | |
| Familiarity | 0.33 | 0.720 |  | **-** | **-** |
| Session Order | -0.88 | 0.053 |  | **-2.305** | **0.0212** |
| Trial Order | -0.10 | 0.801 |  | 0.20 | 0.639 |
| Fluffing (Not zero-inflation) | | | | | |
| Conditional model | | | | | |
| Familiarity | -0.47 | 0.412 |  | -0.08 | 0.891 |
| Session Order | 0.13 | 0.757 |  | -0.48 | 0.064 |
| Trial Order | -0.02 | 0.923 |  | -0.42 | 0.095 |
| Bill wiping | | | | | |
| Conditional model | | | | | |
| Familiarity | 0.60 | 0.353 |  | -0.11 | 0.852 |
| Session Order | **0.97** | **0.049** |  | **1.92** | **<0.001** |
| Trial Order | -0.22 | 0.262 |  | **-0.47** | **0.003** |
| Zero-inflation model | | | | | |
| Familiarity | -2.43 | 0.262 |  |  | |
| Session Order | 0.42 | 0.843 |  | (Not zero inflation) | |
| Trial Order | -1.24 | 0.252 |  |  | |
