# Supplementary material for: The use of artificial songs to assess song recognition in imprinted female songbirds: a concept proposal
Source: Front Psychol. 2024 Sep 4;15:1384794. doi: 10.3389/fpsyg.2024.1384794 (PMC11408183; doi:10.3389/fpsyg.2024.1384794)
Supplement: Supplementary file 2 [file Table_2.DOCX]

Supplementary Material

**Supplementary Table 2.** The results of GLMMs for calling in each subject. The data of JS0327 were analyzed using zero-inflated GLMM, as the data was zero inflation. As the model for JS0327 did not converge with all the explanatory variables, we could include trial order only for its zero-inflation model.

| Session type | Father vs Non-imprinted | |  | Same vs. Different song lineage | |
| --- | --- | --- | --- | --- | --- |
| Fixed Effects | Estimate | *p*-value |  | Estimate | *p-*value |
| JS0311 | | | | | |
| Conditional model | | | | | |
| Familiarity (Non-imprinted) | **-1.97** | **0.007** |  | 38.38 | 1.000 |
| Session Order | **-1.10** | **0.004** |  | -12.64 | 1.000 |
| Trial Order | **-1.46** | **0.005** |  | 19.19 | 1.000 |
| JS0314 | | | | | |
| Conditional model | | | | | |
| Familiarity | **-0.42** | **<0.001** |  | **0.28** | **0.005** |
| Session Order | **1.08** | **<0.001** |  | **0.24** | **0.013** |
| Trial Order | **-0.11** | **0.001** |  | -0.07 | 0.117 |
| JS0327 (Zero-inflation) | | | | | |
| Conditional model | | | | | |
| Familiarity | **-0.81** | **0.016** |  | **1.04** | **<0.001** |
| Session Order | **1.69** | **<0.001** |  | 0.04 | 0.911 |
| Trial Order | -0.04 | 0.875 |  | **-0.72** | **<0.001** |
| Zero-inflation model | | | | | |
| Trial Order | 1.46 | 0.21 |  | -0.48 | 0.641 |
| JS0330 | | | | | |
| Conditional model | | | | | |
| Familiarity | NA | NA |  | NA | NA |
| Session Order | NA | NA |  | NA | NA |
| Trial Order | NA | NA |  | NA | NA |
