# Supplementary material for: The use of artificial songs to assess song recognition in imprinted female songbirds: a concept proposal
Source: Front Psychol. 2024 Sep 4;15:1384794. doi: 10.3389/fpsyg.2024.1384794 (PMC11408183; doi:10.3389/fpsyg.2024.1384794)
Supplement: Supplementary file 3 [file Table_3.DOCX]

Supplementary Material

**Supplementary Table 3.** The results of GLMMs for calls comparing the effect of familiarity between the 1st and 2nd steps of the experiment. The conditional and zero-inflation models were acquired from the summary of the models.

| Fixed Effects | Estimate | *p*-value |
| --- | --- | --- |
| Call | | |
| Conditional model | | |
| **Familiarity (Non-imprinted)** | **-0.37** | **0.050** |
| **Natural vs Artificial song (Natural)** | **-2.46** | **<0.001** |
| **Session Order** | **0.76** | **0.021** |
| **Trial Order** | **-0.18** | **<0.001** |
| Familiarity: Natural vs Artificial | -0.49 | 0.310 |
| Zero-inflation model | | |
| Familiarity (Non-imprinted) | 1.42 | 0..446 |
| Natural vs Artificial song (Natural) | -28.11 | 0.997 |
| Session Order | 1.22 | 0.145 |
| Trial Order | 1.20 | 0.355 |
