# Supplementary material for: The use of artificial songs to assess song recognition in imprinted female songbirds: a concept proposal
Source: Front Psychol. 2024 Sep 4;15:1384794. doi: 10.3389/fpsyg.2024.1384794 (PMC11408183; doi:10.3389/fpsyg.2024.1384794)
Supplement: Supplementary file 4 [file Table_4.DOCX]

Supplementary Material

**Supplementary Table 4.** The list of the subject females and the song stimuli. The mean song duration and mean number of song notes are represented.

| Female | Male Song | Familiarity | Song duration | No. of notes |
| --- | --- | --- | --- | --- |
| JS0311 | **JS0189** | Father | 6.39 | 34.00 |
|  | **JS0170** | Non-imprinted1 | 3.56 | 14.00 |
|  | **JS0219** | Same song lineage | 6.33 | 33.00 |
|  | **JS0102** | Different song lineage | 4.86 | 28.67 |
| JS0314 | **JS0252** | Father | 8.49 | 50.67 |
|  | **JS0194** | Non-imprinted1 | 4.67 | 24.33 |
|  | **JS0016** | Same song lineage | 5.49 | 33.33 |
|  | **JS0072** | Different song lineage | 5.60 | 58.67 |
| JS0327 | **JS0074** | Father | 2.63 | 16.67 |
|  | **JS0231** | Non-imprinted1 | 4.63 | 21.33 |
|  | **JS0040** | Same song lineage | 5.97 | 31.67 |
|  | **JS0170** | Different song lineage | 3.56 | 14.00 |
| JS0330 | **JS0219** | Father | 6.33 | 33.00 |
|  | **JS0102** | Non-imprinted1 | 4.86 | 28.67 |
|  | **JS0064** | Same song lineage | 3.34 | 22.33 |
|  | **JS0100** | Different song lineage | 5.80 | 27.33 |
