# Supplementary material for: The use of artificial songs to assess song recognition in imprinted female songbirds: a concept proposal
Source: Front Psychol. 2024 Sep 4;15:1384794. doi: 10.3389/fpsyg.2024.1384794 (PMC11408183; doi:10.3389/fpsyg.2024.1384794)
Supplement: Supplementary file 6 [file Image_1.pdf]

Supplementary Material

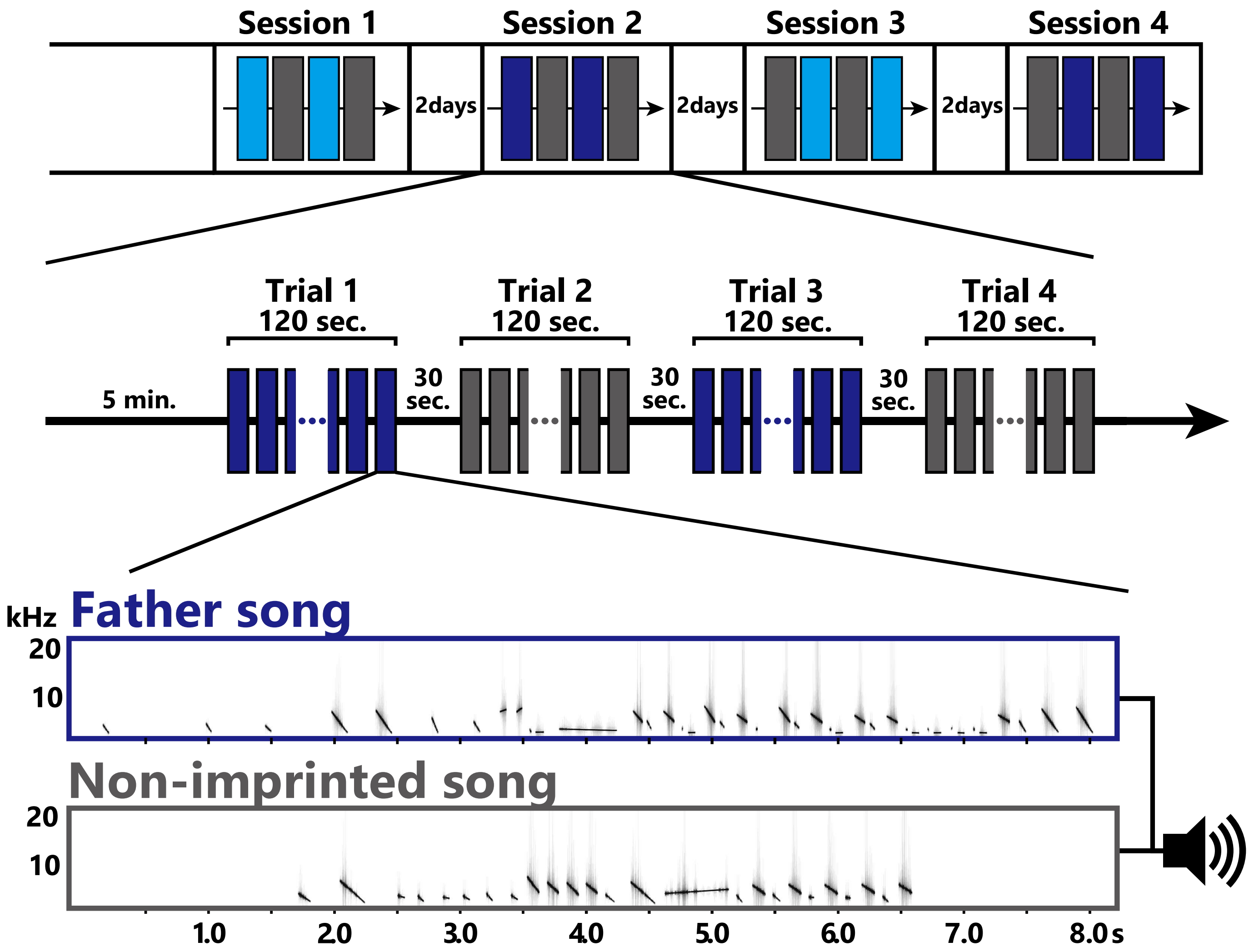

Supplementary Figure 1. Schematic view of the timeline of the experiment. Each subject female experienced a total of four daily sessions scheduled two days apart. Each session was consisted of four 2-min trials that include 12 playbacks of songs (four repetitions of three different songs) from a male (i.e. father or a male that the subject was not imprinted with). The spectrograms show an example of pairwise artificial song stimuli.
