# Supplementary material for: The use of artificial songs to assess song recognition in imprinted female songbirds: a concept proposal
Source: Front Psychol. 2024 Sep 4;15:1384794. doi: 10.3389/fpsyg.2024.1384794 (PMC11408183; doi:10.3389/fpsyg.2024.1384794)
Supplement: Supplementary file 7 [file Image_2.pdf]

Supplementary Material

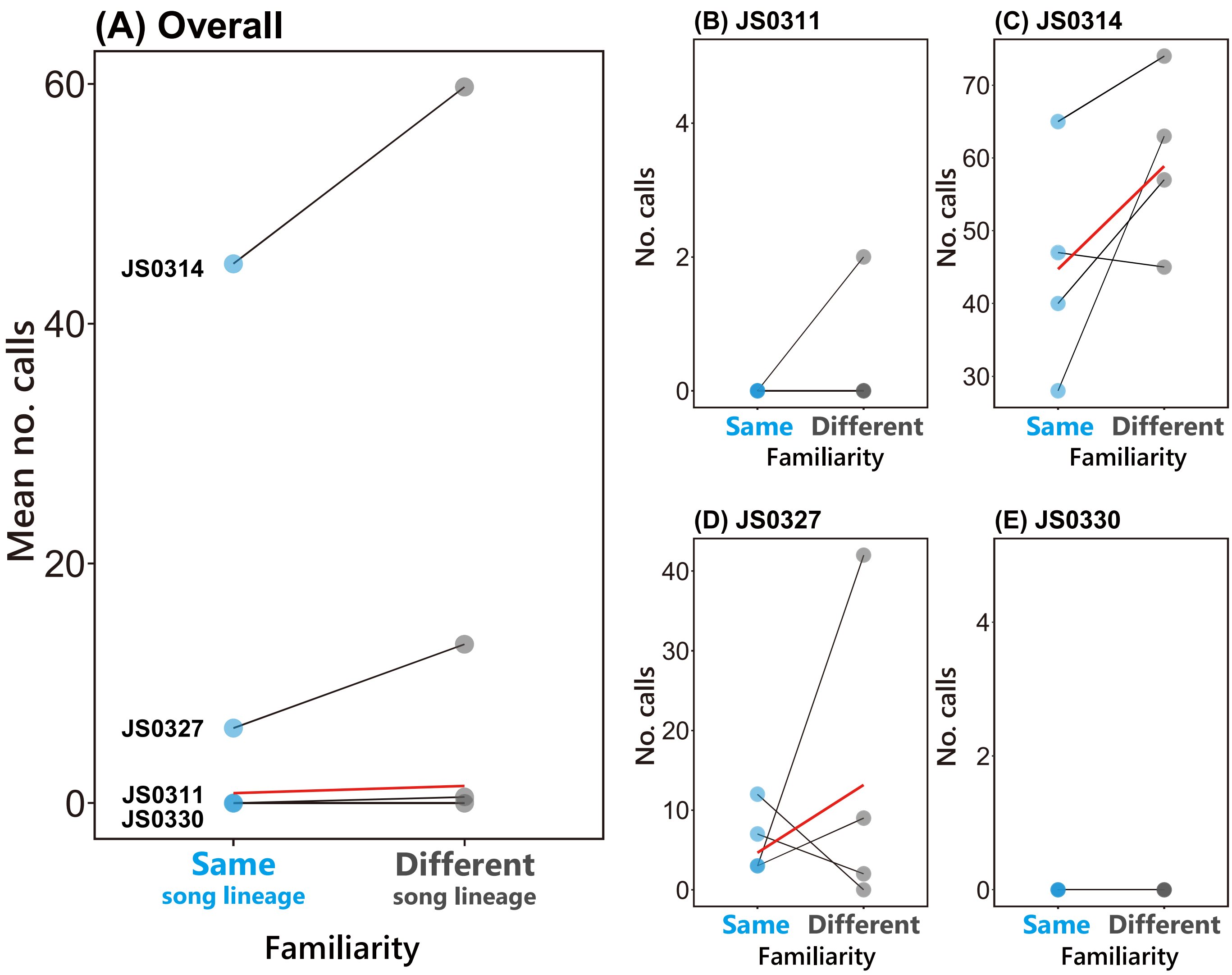

Supplementary Figure 2. The results of calling responses to the same song lineage (non-father) and the different song lineage songs that were synthesized based on natural songs. Unlike the overall non-significant tendency for female Java sparrows to prefer the same song lineage songs (see Lewis et al. 2024), the subject females of this study did not show the same pattern, instead responding more to the different lineage songs. In line with this, subject females showed preference for different lineage songs in the synthesized song playback tests here. The results of calling responses to the artificial same and same lineage songs. (A) shows the average number of calls per trial shown by four subject females, where each connected dots correspond to each subject, and the red line indicates the effect of the model estimating the statistically significant difference. (B)-(E) show calling responses shown by each subject in each trial, where line connects the continuing order trials.
