# Supplementary material for: The use of artificial songs to assess song recognition in imprinted female songbirds: a concept proposal
Source: Front Psychol. 2024 Sep 4;15:1384794. doi: 10.3389/fpsyg.2024.1384794 (PMC11408183; doi:10.3389/fpsyg.2024.1384794)
Supplement: Supplementary file 8 [file Image_3.pdf]

Supplementary Material

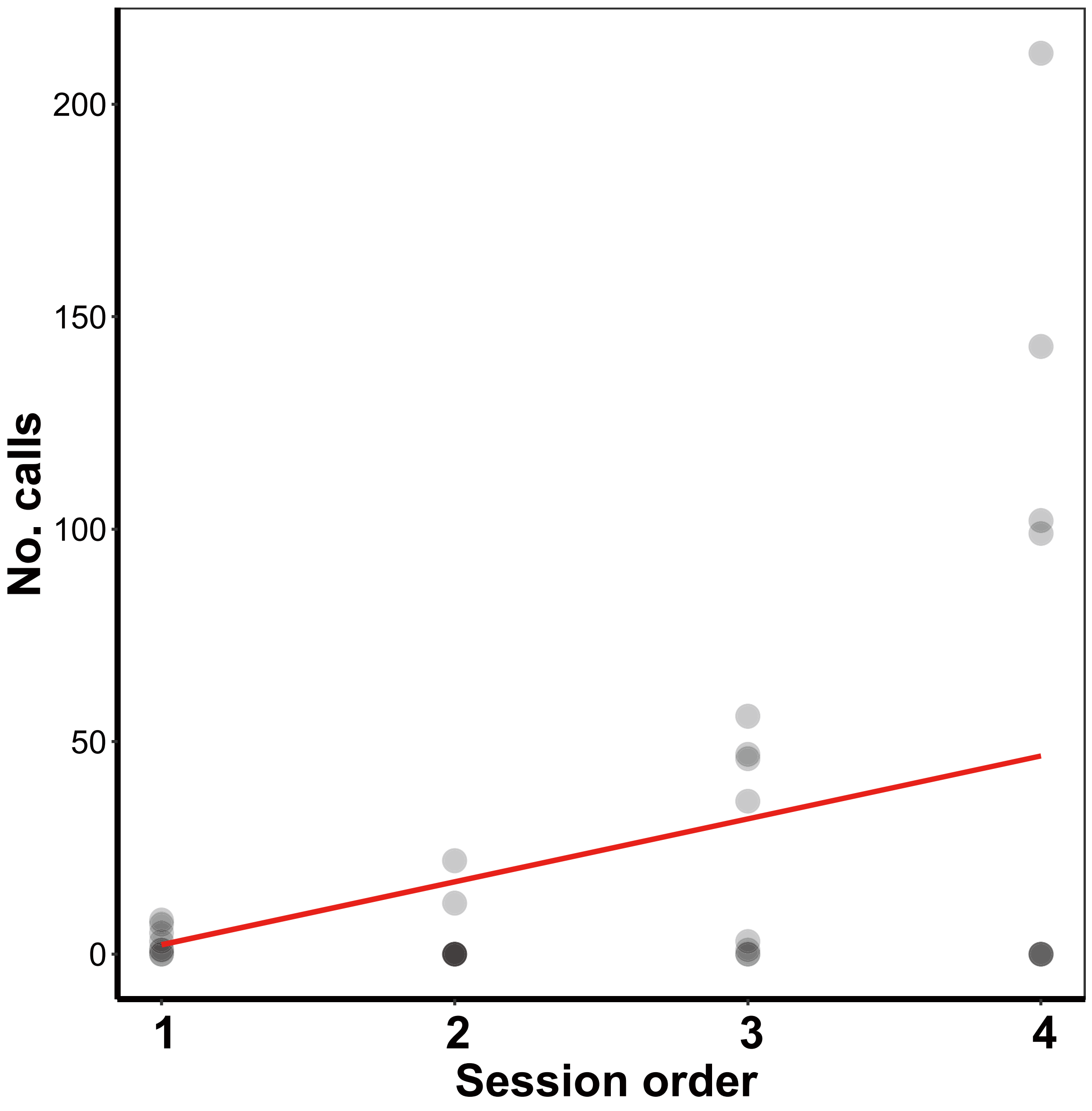

Supplementary Figure 3. Each point indicates the number of calling responses in each trial. The red line indicates the effect of the session order.
