# Supplementary material for: The use of artificial songs to assess song recognition in imprinted female songbirds: a concept proposal
Source: Front Psychol. 2024 Sep 4;15:1384794. doi: 10.3389/fpsyg.2024.1384794 (PMC11408183; doi:10.3389/fpsyg.2024.1384794)
Supplement: Supplementary file 9 [file Image_4.pdf]

Supplementary Material

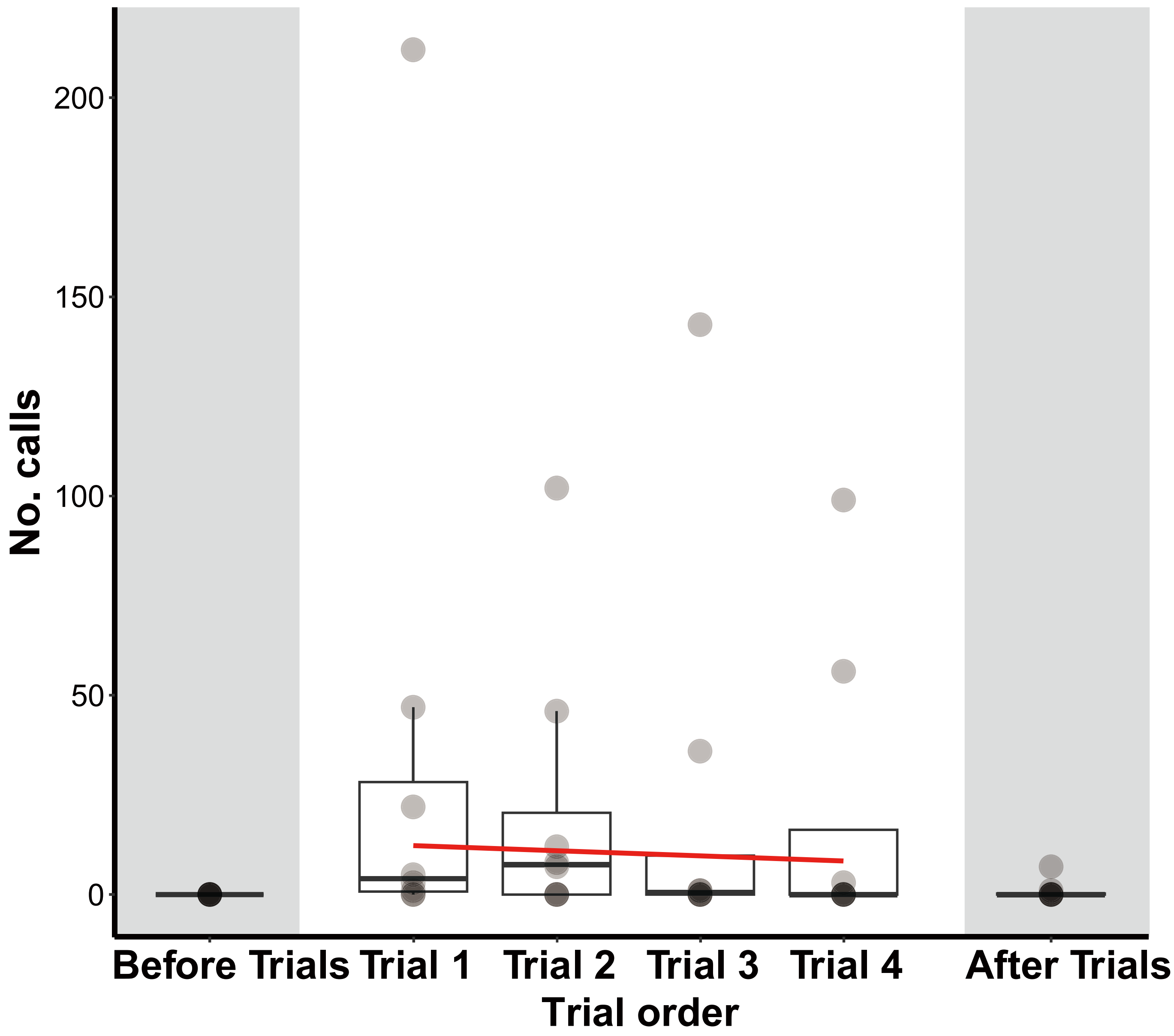

Supplementary Figure 4. Plots show changes in the number of calls depending on trial order compared with 2-min control period, which was before/after each session. The red line indicates the effect of the trial order. Calling responses were shown in each trial, and few were shown before and after trials.
