# Supplementary material for: The use of artificial songs to assess song recognition in imprinted female songbirds: a concept proposal
Source: Front Psychol. 2024 Sep 4;15:1384794. doi: 10.3389/fpsyg.2024.1384794 (PMC11408183; doi:10.3389/fpsyg.2024.1384794)
Supplement: Supplementary file 10 [file Image_5.pdf]

Supplementary Material

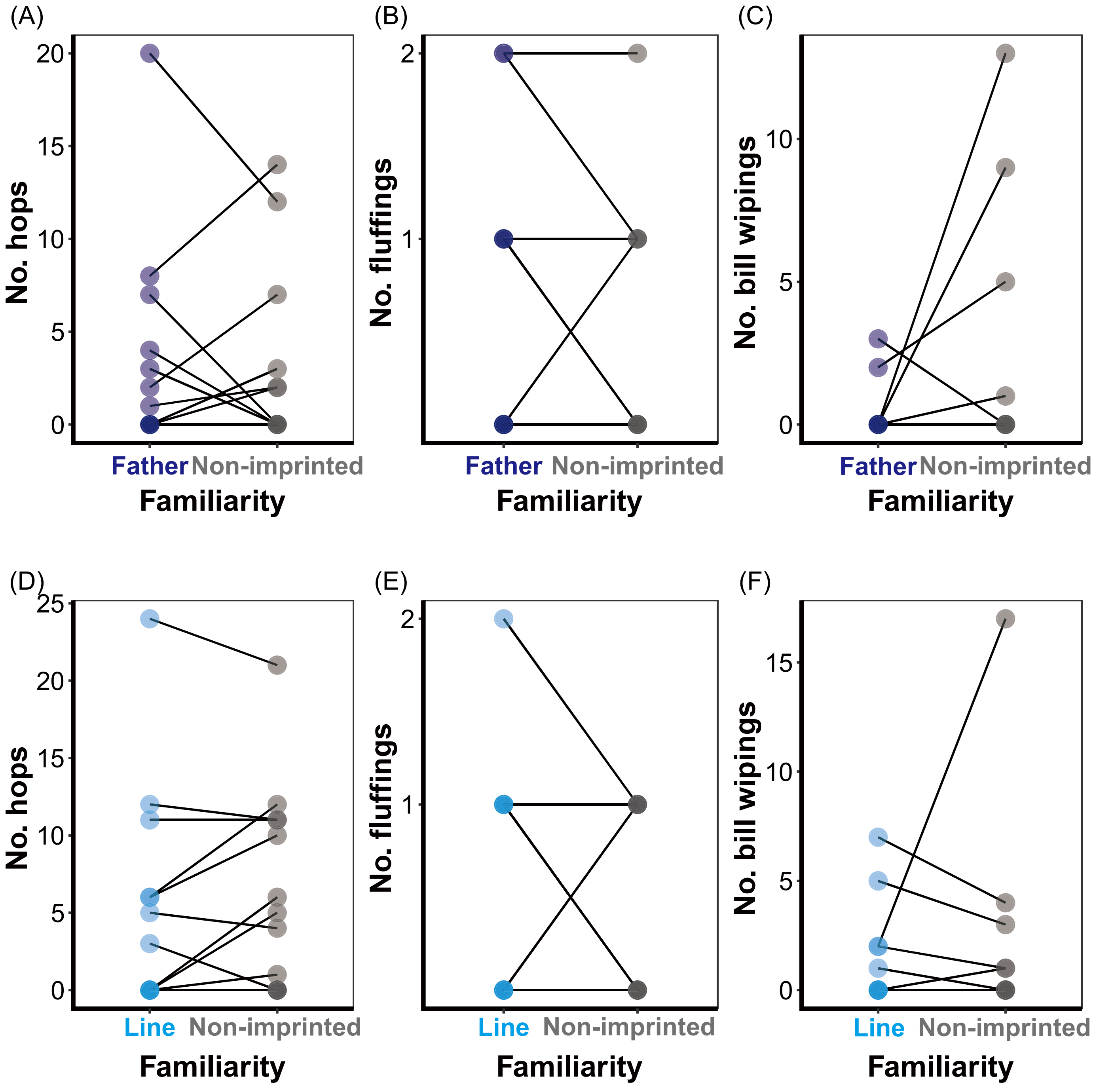

Supplementary Figure 5. The results of hopping, fluffing, and bill-wiping responses to the father vs. non-imprinted songs (A - C), and line (the same song lineage non-father) vs. non-imprinted songs (D - F) in the 2nd step experiment. Each connected points indicats the number of behaviors (A, D: hops; B, E: fluffings; C, F: bill wipings), and each line connects the continuing order trials.
